# Supplementary material for: Increased Susceptibility of Mycobacterium tuberculosis to Ethionamide by Expressing PPs-Induced Rv0560c
Source: Antibiotics (Basel). 2022 Oct 4;11(10):1349. doi: 10.3390/antibiotics11101349 (PMC9598822; doi:10.3390/antibiotics11101349)
Supplement: Supplementary file 1 [file antibiotics-11-01349-s001.zip › antibiotics-1935178-supplementary.pdf]

Supplementary Information

Supplementary method

Synthesis of Rv3855 protein

Synthesis of Rv3855 protein was performed in Young In Frontier (Seoul, Korea) by referring to NCBI gene information (Gene symbol, *ethR*; Gene description, HTH-type transcriptional repressor EthR; Locus tag, Rv3855, Sequence: NC_000962.3 (4327549..4328199)) using gDNA of *M. tuberculosis* H37Rv as a template. (Supplementary Fig. S5). For the convenience of purification, cloning was performed using the pET23b vector with C-terminal 6×His-tag. In this step, restriction enzyme (New England Biolabs, USA), InnuPREP Gel extraction kit (Analytik Jena, Germany), T4 DNA ligase (Promega, USA), and plasmid mini kit (NucleoGen, Korea) were used. After digesting the synthesized DNA with NdeI/XhoI, it was ligated with a pET23b vector (NdeI/XhoI). Cloning was completed as the DNA insert was confirmed (Supplementary Fig. S5A).

To confirm the optimal expression and purification conditions, the target protein's expression level, solubility, and column binding affinity were tested on a small scale.

Expression was tested under the following conditions using four cell strains as hosts (Supplementary Fig. S5B). Two culture conditions (37℃, 200 rpm, 4 hrs; 20℃, 110 rpm, over-night,) and two lysis buffers (buffer A: 20 mM Tris, 10 mM NaCl, pH 8.0; buffer B: 50 mM NaH_2PO_4, 300 mM NaCl, and pH 8.0) were applied. OD_{600nm} was 0.4~0.7. The final IPTG concentration was 0.4 mM. LB media (Amresco, USA), IPTG (LPS solution, Korea), and Ni-NTA Agarose (Qiagen, USA) were used for this step. Based on this result, a native binding test was performed with BL21 (DE3) pLysS cell lysate (buffer A, B) cultured at 37℃.

The Ni-NTA affinity binding test was performed with buffers A and B. As a result, the

purity of the target protein was better when native condition was applied in buffer A for purification (Supplementary Fig. S5C). BL21 (DE3) pLysS was used as the host strain. The culture temperature was 37°C.

After binding His tagging protein using Ni-NTA resin, the target protein was separated by eluting the tagged protein according to the concentration of imidazole (Supplementary Fig. S5D). BL21 (DE3) pLysS was used for large-scale culture (OD_{600nm}=0.4; 0.4mM IPTG, 200 rpm, 4hr, 37°C, 3L). Harvested cells were used for protein purification. Compositions of the wash buffer and elution buffer were the same (1× PBS, 10 mM NaCl, pH 7.4) except for the imidazole concentration.

Amicon Ultra-15 Centrifugal Filter Unit (Merck, USA) wan used for concentration of protein. The concentration of proteins was measured with the Bradford assay. Protein purification was confirmed by SDS-polyacrylamide gel electrophoresis (SDS-PAGE) (Supplementary Fig. S2E). Finally, 25 mg of protein was synthesized (1.5 mg/ml).

Synthesis of Rv2887 protein

Young In Frontier (Seoul, Korea) performed the synthesis of Rv2887 protein based on NCBI gene information (Gene symbol, *Rv2887*; Gene description, HTH-type transcriptional regulator; Locus tag, Rv2887, Sequence: NC_000962.3 (3196431 to 3196850)) using gDNA of *M. tuberculosis* H37Rv as a template (Supplementary Fig. S6). The overall protein synthesis proceeded similarly to the protein synthesis process described above.

Digestion of the synthesized DNA was performed using NdeI/XhoI. Digested DNA was then ligated with a pColdII vector (NdeI/XhoI) (Supplementary Fig. S6A). Expression was conducted using the four cell strains mentioned above as hosts (Supplementary Fig. S6B). The final IPTG concentration was 0.4 mM. Cells were cultured overnight at 15°C and 110 rpm to reach an OD_{600} nm of 0.4 to 0.7. Two lysis buffers (buffers A and B) were used. Based

on this result, a native binding test was performed with BL21 (DE3) pLysS cell lysate (buffers A and B) cultured at 15°C.

The Ni-NTA affinity binding test was performed using buffers A and B. As a result of the experiment, the purity of the target protein was better when the purification was performed under the native condition of buffer B (Supplementary Fig. S6C). BL21 (DE3) pLysS was used as the host strain, and the culture temperature was set at 15°C. Buffers A and B used for the binding, wash, and elution buffers were the same as those used for Rv3885 synthesis.

After binding His tagging protein using Ni-NTA resin, the target protein was separated by eluting the tagged protein according to the concentration of imidazole (Supplementary Fig. S6D). BL21 (DE3) pLysS was used for large-scale culture (OD_{600nm} = 0.4; 0.4 mM IPTG, 100 rpm, overnight, 37°C, 3L). Harvested cells were used for purification (flow rate = 0.5 ml/min). Buffer A was used as the lysis buffer. Compositions of the wash buffer and elution buffer were the same as the lysis buffer except for the imidazole concentration.

After the protein concentration process, the synthesis of 16 mg of protein (3.2 mg/ml) was completed. It was confirmed by SDS-PAGE (Supplementary Fig. S3E).

Synthesis of Rv0560c protein

Young In Frontier (Seoul, Korea) performed the synthesis of Rv0560c protein based on NCBI gene information (Gene symbol, *Rv0560c*; Gene description, benzoquinone methyltransferase; Locus tag, Rv0560c, Sequence: NC_000962.3 (650779 to 651504, complement)) using gDNA of *M. tuberculosis* H37Rv as a template (Supplementary Fig. S7). The overall protein synthesis proceeded similarly to the protein synthesis process described above.

Digestion of the synthesized DNA used NdeI/XhoI. Digested DNA was ligated with a pET23b vector (NdeI/XhoI) (Supplementary Fig. S7A). One mutation was identified. It was

a silent mutation without changing the amino acid. Optimization of expression was the same as described for Rv3855 protein synthesis (Supplementary Fig. S7B).

Based on this result, an affinity binding test was performed successively using the BL21(DE3) strain (Supplementary Fig. S7C). Two types of binding buffers, wash buffers, and elution buffers were used: binding buffer (buffer A; buffer C, 8 M urea, 50 mM NaH_2PO_4, 300 mM NaCl, and pH 8.0), wash buffer (buffers A and C containing 20 mM imidazole), elution buffer (buffers A and C containing 100/250 mM). Buffer A provided a native condition, and buffer C provided a natural condition.

According to the above results, Ni-NTA affinity purification was performed under denaturing conditions (Supplementary Fig. S7D). BL21 (DE3) strain was used for large-scale culture (OD_{600nm} = 0.5; 0.4 mM IPTG, 200 rpm, 4 hr, 37°C, 3L). Harvested cells were used for protein purification. Denaturing buffer (buffer C) was used. Compositions of the wash buffer and elution buffer were the same as those of the denaturing buffer except for the imidazole concentration.

After the protein concentration process, the synthesis of 10 mg of protein (1 mg/ml) was completed. It was confirmed by SDS-PAGE (Supplementary Fig. S4E).

Preparation of rabbit polyclonal antisera against Rv0560c protein

Polyclonal antiserum against Rv0560c protein was prepared by Young In Frontier (Seoul, Korea). The synthesized Rv0560c protein was used for immunization. Male 4-6 weeks old New Zealand white rabbits were used. Detailed immunization tables are presented in Supplementary Table S2.

Microarray

The overall microarray experiments with drugs in *M. tuberculosis* were performed according to previously reported methods [2]. First, RNA from *M. tuberculosis* was extracted with the

RNAprotect Bacteria Reagent kit (QIAGEN, Germany). The extracted RNA was sent to ebiogen (Seoul, Korea) for microarray analysis. Upon arrival, RNA samples were checked for quality using a bioanalyzer (Agilent, USA). It was confirmed that $OD_{260/280}$ was > 1.8 and $OD_{260/230}$ was > 1.6, and degradation was evaluated by migration pattern.

A cDNA synthesis kit (Bio-Rad, USA) and a Low Input Quick Amp WT Labeling Kit (Agilent, USA) were used for reverse transcription of RNA into cDNA and subsequent synthesis and hybridization of the target cRNA probe, respectively. After evaluating the labeling efficiency, the fragmented cRNA solution was resuspended in hybridization buffer and then injected into the assembled microarray for *M. tuberculosis* (MYcroarray.com, 3×20k). Arrays were hybridized for 17 h in a hybridization oven (Agilent, USA) at 57°C. The hybridized microarrays were analyzed with an Agilent DNA microarray scanner (Agilent, USA). Feature Extraction software (Agilent, USA) was used for quantification.

Differentially transcribed genes were sorted by functional category [3]. A hypergeometric distribution was used to determine which set of genes in a particular functional category responded significantly to the drug [4, 5].

2DE

Control and drug-treated bacterial cells were harvested, lysed in cell lysis buffer (pH 7, 0.3% SDS, 28 mM Tris base), and transferred to a tube containing silica beads (0.1 mm, MP biomedicals, USA). These bacterial cells were disrupted with 10 pulses for 30 s using a BeadBug microtube homogenizer (Benchmark Scientific, Inc., USA) at 4,000 Hz with medium ice-cooling for 20 s. The concentration of the extracted protein was measured by BCA (bicinchoninic acid) assay (Pierce, USA). 2DE experiment was performed by ProteomeTech (Seoul, Korea) as previously described [6].

Briefly, harvested protein samples were suspended in 0.5 ml of 50 mM Tris buffer, then

the lysate was homogenized and then collected by centrifugation. IPG strips (pH 3-10 NL, Amersham Biosciences, USA) were rehydrated in swelling buffer, where protein lysates were loaded using a Multiphor II apparatus (Amersham Biosciences, USA) at 57 kVh. 2-DE separation was performed on SDS-polyacrylamide gels, the gel was fixed in a solution (40% methanol, 5% phosphoric acid) for 1 h, and the gel was stained with Colloidal Coomassie Brilliant Blue solution (G-250, ProteomeTech, Korea) for 5 h. Gels were destained with water, images were acquired using Image Scanner III (GE Healthcare, USA), and protein spots were analysed by ImageMaster 2D Platinum software (Amersham Biosciences, USA). As reported previously, the separation of proteins from the gel and identification was performed [6, 7].

Table S1. Immunization schedule for the preparation of polyclonal rabbit anti-Rv0560c serum

Time	Description
Week 0 (pre-immune bleeding)	Pre-immune serum collection (ear vain)
Week 0 (pre-immunize)	0.5mg/Rabbit in Complete Freund's Adjuvant (CFA) (subcutaneous injection)
Week 4 (1st immunize)	200 μg/Rabbit in Incomplete Freund's Adjuvant (IFA) (subcutaneous injection)
Week 5 (1st bleed)	1st production bleed (ear vain)<br>1st ELISA test
Week 6 (2nd immunize)	200 μg/Rabbit in Incomplete Freund's Adjuvant (IFA) (subcutaneous injection)
Week 7 (2nd bleed)	2nd production bleed (ear vain)<br>2nd ELISA test
Week 8 (3rd immunize)	200 μg/Rabbit in Incomplete Freund's Adjuvant (IFA) (subcutaneous injection)
Week 9 (final bleed)	Sacrifice (Heart puncture) Final ELISA test

Table S2. Number of genes (fold change cutoff: > 2) in *M. tuberculosis* H37Rv regulated by treatment with PPs at 1× and 10× MIC for 6 hours at 37 °C arranged by functional classification

	PP1S				PP2S			
	1× MIC		10× MIC		1× MIC		10× MIC	
Functional classification	up	down	up	down	up	down	up	down
Ⅰ. Small-molecule metabolism								
A. Degradation								
10. Carbon compounds (22)[d]	0	0	0	0	0	4[a]	0	1
20. Amino acids and amines (18)	0	0	0	0	0	0	0	0
30. Fatty acids (119)	1	4	1	3	0	5	1	0
40. Phosphorous compounds (4)	0	0	0	0	0	0	0	0
B. Energy Metabolism								
10. Glycolysis (12)	0	0	0	0	0	0	0	0
20. Pyruvate dehydrogenase (6)	0	0	0	0	0	0	0	0
30. TCA cycle (19)	0	0	1	0	0	0	0	0
40. Glyoxylate bypass (5)	0	0	0	0	0	0	0	0
50. Pentose phosphate pathway (11)	0	0	0	0	0	0	0	0
60. Respiration								
a. aerobic (30)	0	1	1	1	0	0	0	0
b. anaerobic (15)	0	0	0	0	0	1	0	0
c. Electron transport (15)	0	0	0	0	0	1	0	0
70. Miscellaneous oxidoreductases and oxygenases (171)	2	2	3	5	0	6	0	1
80. ATP-proton motive force (8)	0	0	0	0	0	0	0	0
C. Central intermediary metabolism								
10. General (13)	0	1	0	0	0	0	0	0
20. Gluconeogenesis (2)	0	0	0	0	0	0	0	0
30. Sugar nucleotides (14)	0	0	0	0	0	0	0	0
40. Amino sugars (1)	0	0	0	0	0	0	0	0
50. Sulphur metabolism (15)	0	0	0	1	0	0	0	0
D. Amino acid biosynthesis								
10. Glutamate family (19)	0	1	0	0	0	0	0	0
20. Aspartate family (21)	0	0	0	0	0	0	0	0
30. Serine family (15)	0	0	0	0	0	0	0	0
40. Aromatic amino acid family (15)	0	0	0	0	0	0	0	0
50. Histidine (11)	0	0	0	0	0	0	1[a]	0
60. Pyruvate family (1)	0	0	0	1	0	0	0	0
70. Branched amino acid family (13)	0	0	0	0	0	0	0	0
E. Polyamine synthesis (1)	0	0	0	0	0	0	0	0
F. Purines, pyrimidines, nucleosides, and nucleotides								
10. Purine ribonucleotide biosynthesis (20)	0	0	0	0	0	1	0	0
20. Pyrimidine ribonucleotide biosynthesis (9)	0	0	0	0	0	0	0	0
30. 2'-deoxyribonucleotide metabolism (12)	0	0	0	0	0	0	0	0
40. Salvage of nucleosides and nucleotides (10)	0	0	0	0	0	0	0	0
50. Miscellaneous nucleoside/nucleotide reactions (9)	0	0	0	0	0	0	0	0
G. Biosynthesis of cofactors, prosthetic groups, and carriers								
10. Biotin (8)	0	0	0	0	0	1	0	0
20. Folic acid (11)	0	0	0	0	0	0	0	0
30. Lipoate (2)	0	0	0	0	0	0	0	0
40. Molybdopterin (20)	0	0	0	0	0	1	0	0
50. Pantothenate (4)	0	0	0	0	0	0	0	0
60. Pyridoxine (1)	0	0	0	0	0	0	0	0
70. Pyridine nucleotide (4)	0	0	0	0	0	0	0	0
80. Thiamine (4)	0	0	0	0	0	0	0	0
90. Riboflavin (8)	1	0	0	1	0	0	0	0
100. Thioredoxin, glutaredoxin, and mycothiol (8)	0	0	0	0	0	0	0	0
110. Menaquinone, PQQ, ubiquinone, and other terpenoids (15)	1	1	1	1	0	1	0	0
120. Heme and porphyrin (9)	0	0	0	0	0	0	0	0
130. Cobalamin (17)	0	1	0	1	0	0	0	0
140. Iron utilization (6)	0	0	0	0	0	0	0	0
H. Lipid Biosynthesis								
10. Synthesis of fatty and mycolic acids (26)	0	0	0	0	0	1	0	0
20. Modification of fatty and mycolic acids (14)	0	0	0	0	0	0	0	0
30. Acyltransferases, mycoloyltransferases, and phospholipid synthesis (25)	0	0	0	0	0	0	0	0
I. Polyketide and non-ribosomal peptide synthesis (41)	1	1	1	1	0	0	0	0
J. Broad regulatory functions								
10. Repressors/activators (143)	3	3	4[a]	5	0	7	1	0
20. Two-component systems (30)	0	2	0	1	0	4[a]	0	0
30. Serine-threonine protein kinases and phosphoprotein phosphatases (14)	0	0	0	0	0	0	0	0
Ⅱ. Macromolecule metabolism								

A. Synthesis and modification of macromolecules								
10. Ribosomal protein synthesis and modification (56)	0	0	0	0	0	2	0	0
20. Ribosome modification and maturation (3)	0	0	0	0	0	0	0	0
30. Aminoacyl tRNA synthases and their modification (26)	0	0	0	0	0	0	0	0
40. Nucleoproteins (4)	0	0	0	0	0	0	0	0
50. DNA replication, repair, recombination, and restriction/modification (69)	0	2	0	1	0	2	1	1
60. Protein translation and modification (15)	0	0	0	2[a]	0	1	0	0
70. RNA synthesis, RNA modification, and DNA transcription (32)	0	0	0	0	0	1	0	1
80. Polysaccharides (cytoplasmic) (8)	0	0	0	0	0	0	0	0
B. Degradation of macromolecules								
10. RNA (6)	0	0	0	0	0	0	0	0
20. DNA (3)	0	0	0	0	0	0	0	0
30. Proteins, peptides, and glycopeptides (34)	0	0	0	0	0	1	0	0
40. Polysaccharides, lipopolysaccharides, and phospholipids (8)	0	0	0	0	0	1	0	0
50. Esterases and lipases (27)	0	0	0	1	0	3	1[b]	0
60. Aromatic hydrocarbons (9)	0	0	0	0	0	2	0	0
C. Cell envelope								
10. Lipoproteins(lppA-lpr0) (65)	0	2	0	3	0	6[a]	0	0
20. Surface polysaccharides, lipopolysaccharides, proteins, and antigens (38)	0	1	0	0	0	4	1[a]	2[b]
30. Murein sacculus and peptidoglycan (28)	0	0	0	0	0	1	0	0
40. Conserved membrane proteins (17)	0	0	1	0	0	1	0	0
50. Other membrane proteins (210)	4	2[a]	3	5	0	12	1[a]	0
Ⅲ. Cell processes								
A. Transport/binding proteins								
10. Amino acids (17)	0	2[a]	0	2	0	1	0	0
20. Cations (31)	0	1	0	1	0	1	0	0
30. Carbohydrates, organic acids, and alcohols (19)	0	0	0	0	0	4[b]	0	0
40. Anions (34)	0	0	0	0	0	1	0	0
50. Fatty acid transport (2)	0	0	0	0	0	0	0	0
60. Efflux proteins (20)	0	0	0	0	0	0	0	0
B. Chaperones/heat shock (16)	0	0	0	2	0	0	0	1[a]
C. Cell division (19)	0	0	0	0	0	0	0	0
D. Protein and peptide secretion (14)	0	0	0	0	0	0	0	0
E. Adaptations and atypical conditions (11)	0	0	0	0	0	0	0	0
F. Detoxification (22)	0	1	0	0	0	1	0	0
Ⅳ. Other								
A. Virulence (38)	0	0	0	0	0	2	0	0
B. IS elements, repeated sequences, and phage								
10. IS elements								
a. IS6110 (32)	0	0	0	0	0	0	0	0
b. IS1081 (6)	0	0	0	0	0	3[b]	0	0
c. Others (52)	0	2	0	3	0	5	0	0
20. REP13E12 family (10)	0	0	0	0	0	3[b]	0	0
30. Phage-related functions (34)	0	6[c]	1	3[a]	0	4	0	0
C. PE and PPE families								
10. PE family								
a. PE subfamily (36)	1	2	0	0	0	3	1[b]	0
b. PE_PGRS subfamily (58)	0	4[a]	1	6[b]	0	1	0	0
20. PPE family (68)	0	8[c]	0	3	0	8[b]	0	0
D. Antibiotic production and resistance (14)	0	0	0	0	0	1	1[a]	0
E. Bacteriocin-like proteins (3)	0	0	1	0	0	0	0	0
F. Cytochrome P450 enzymes (22)	0	0	0	0	0	3	0	0
G. Coenzyme F420-dependent enzymes (3)	0	0	0	0	0	0	0	0
H. Miscellaneous transferases (61)	2	1	1	1	0	0	1	0
I. Miscellaneous phosphatases, lyases, and hydrolases (18)	0	1	0	0	0	1	0	0
J. Cyclases (6)	0	0	0	0	0	0	0	0
K. Chelatases (2)	0	0	0	0	0	0	0	0
Ⅴ. Conserved hypotheticals (914)	8	15	11	14	0	25[b]	1	1
Ⅵ. Unknowns (588)	4	17	3	23	0	35[a]	1	2[b]

[d]Numbers in parentheses indicate the total number of genes in the *M. tuberculosis* H37Rv genome in the category.

Significance was determined using the hypergeometric distribution function ($^{a}p < 0.05$, $^{b}p < 0.01$, $^{c}p < 0.001$).

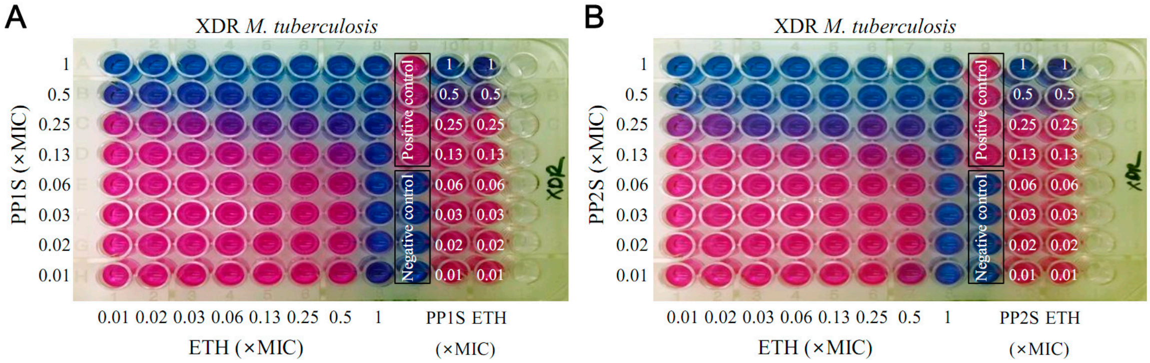

Figure S1. In vitro evaluation of the effect of PPs on the susceptibility of XDR *M. tuberculosis* to ethionamide (ETH). Whether (A) PP1S or (B) PP2S could increase the anti-tuberculous activity of ETH was tested against the XDR strain. In the presence of PPs at 0.5 × MIC, ETH inhibited the growth of *M. tuberculosis* even at concentrations much lower than 1× MIC.

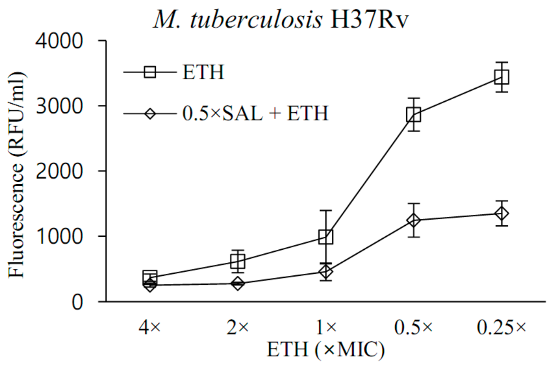

Figure S2. Effect of combining ethionamide (ETH) with salicylate (SAL) on *M. tuberculosis* H37Rv. The growth profile of the H37Rv strain treated with ETH in combination with SAL was assessed by resazurin assay. The results showed a significant increase in the susceptibility of *M. tuberculosis* H37Rv to ETH below 1× MIC in the presence of 0.5× MIC of SAL ($p < 0.05$). Values were expressed as mean ± standard deviation, and statistical significance was determined using an unpaired Student's t-test by comparing the fluorescence values of samples treated with ETH.

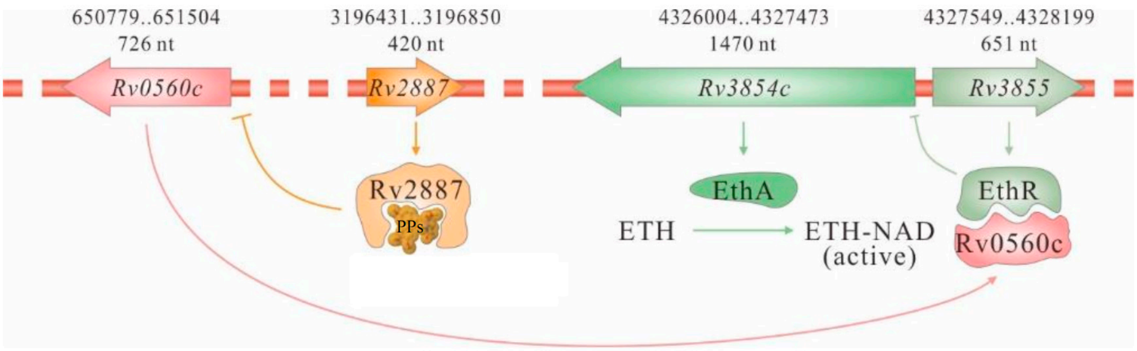

Figure S3. Rv2887-Rv0560c-EthR-EthA pathway. The expression of Rv0560c is caused by the interaction of its repressor, Rv2887, with PPs. Moreover, Rv0560c interacts with EthR, a repressor of EthA (ethionamide bioactivator), which functions as an ethionamide (ETH) bioactivator in *M. tuberculosis*.

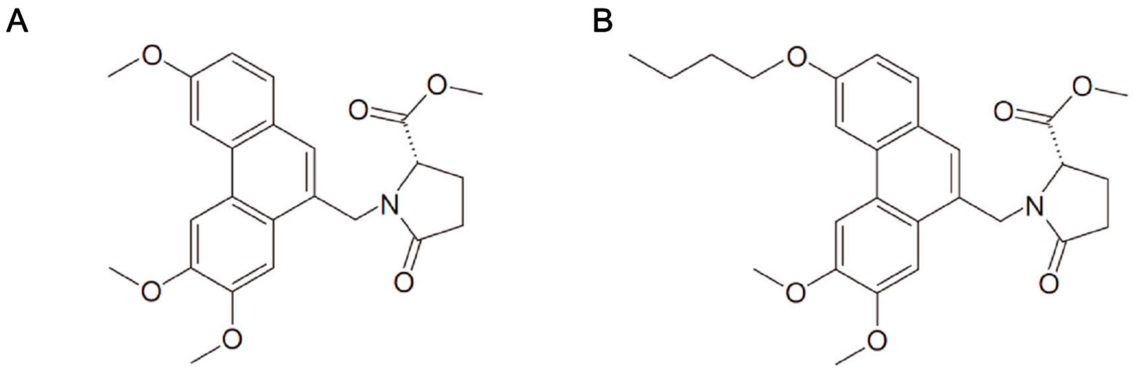

Figure S4. Chemical structure of PPs. (A) PP1S. (B) PP2S.

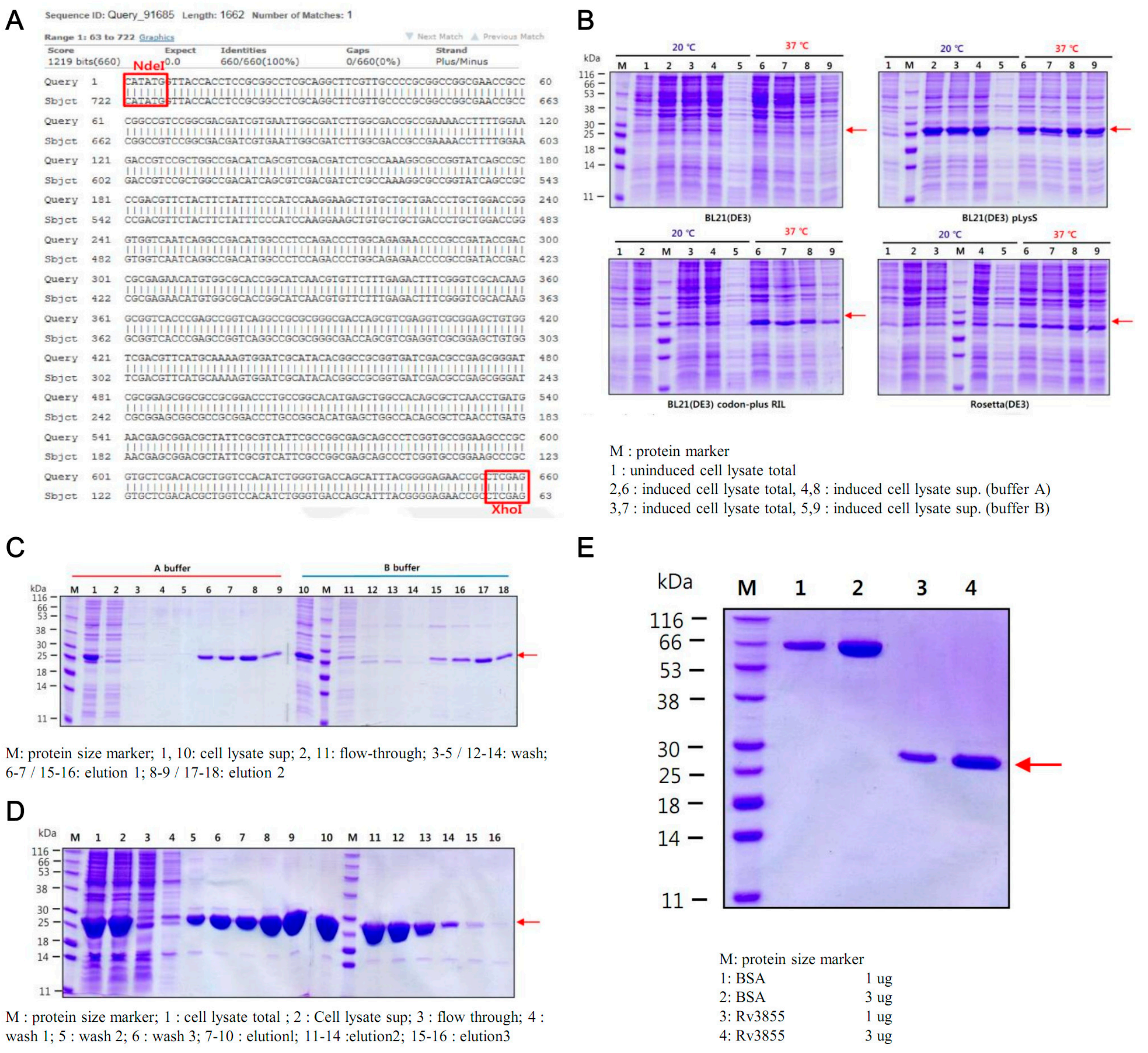

Figure S5. Synthesis of Rv3855 protein. (A) A DNA fragment corresponding to the *Rv3855* gene amplified using the H37Rv gDNA as a template was inserted into the pET23b vector using NdeI/XhoI. (B) Expression was tested using BL21 (DE3), BL21 (DE3) pLysS, BL21 codon-plus RIL, and Rosetta (DE3) as hosts. Based on the expression results, a natural binding test was performed using BL21 (DE3) pLysS cell lysates (buffers A, B) cultured at 37℃. (C) Ni-NTA affinity binding test was performed using buffers A and B. As a result of the experiment, the purity of the target protein was better when purification was performed in buffer A. (D) After binding His tagging protein using Ni-NTA resin, the target protein was isolated by eluting the tagging protein according to the concentration of imidazole. (E) Harvested cells were purified and concentrated. SDS-PAGE confirmed the finally synthesized protein.

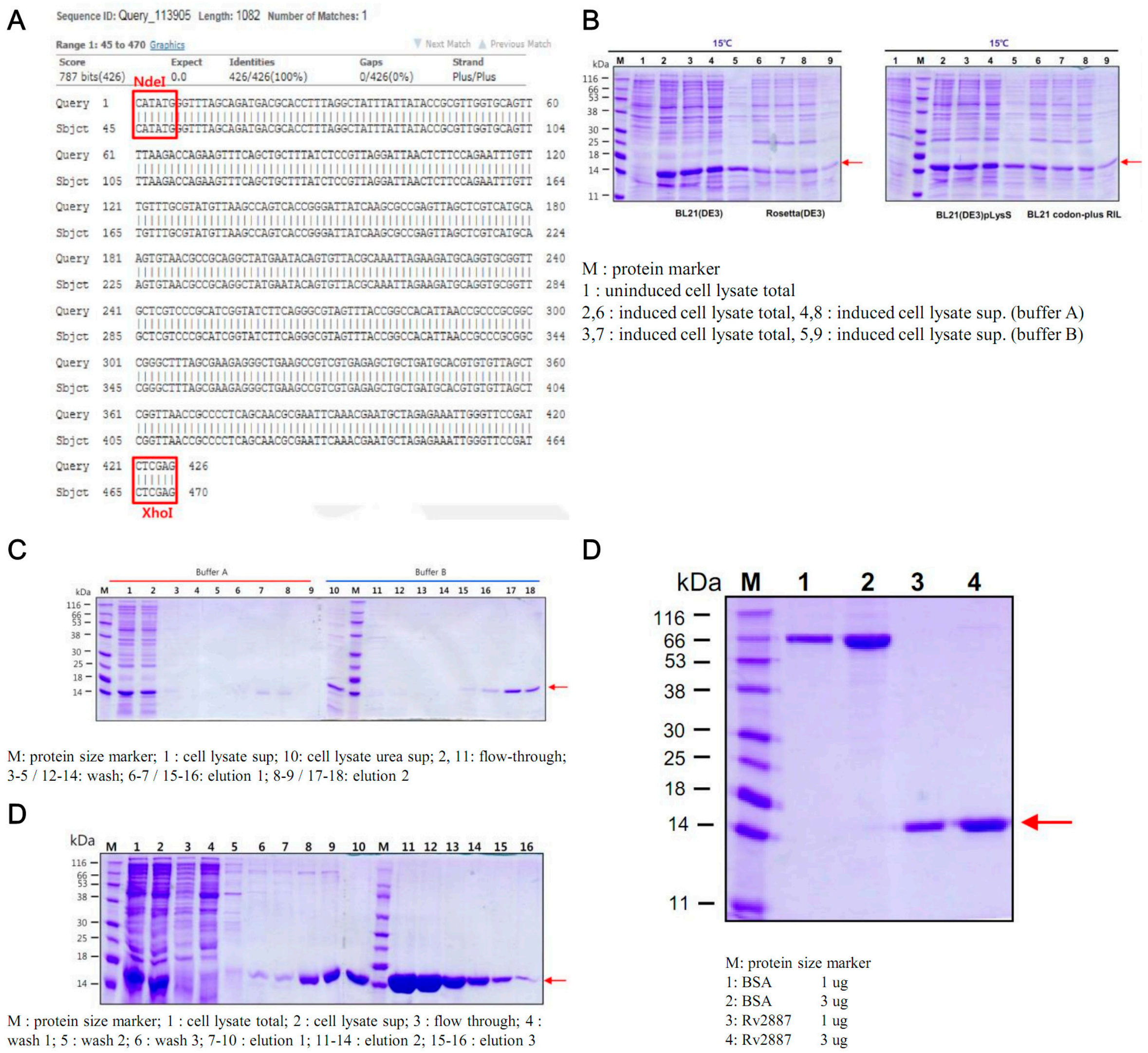

Figure S6. Synthesis of Rv2887 protein. (A) A DNA fragment corresponding to the *Rv2887* gene amplified using H37Rv gDNA as a template was inserted into the pColdII vector using NdeI/XhoI. (B) Based on expression test results, a natural binding test was performed using a BL21 (DE3) pLysS cell lysate cultured at 15°C. (C) Ni-NTA affinity binding test was performed under two buffer conditions. As a result of the experiment, the purity of the target protein was superior when the protein was purified with buffer B. (D) After binding His tagging protein using Ni-NTA resin, the target protein was isolated by eluting with different concentrations of imidazole. (E) Harvested cells were purified and concentrated, and SDS-PAGE confirmed the finally synthesized protein.

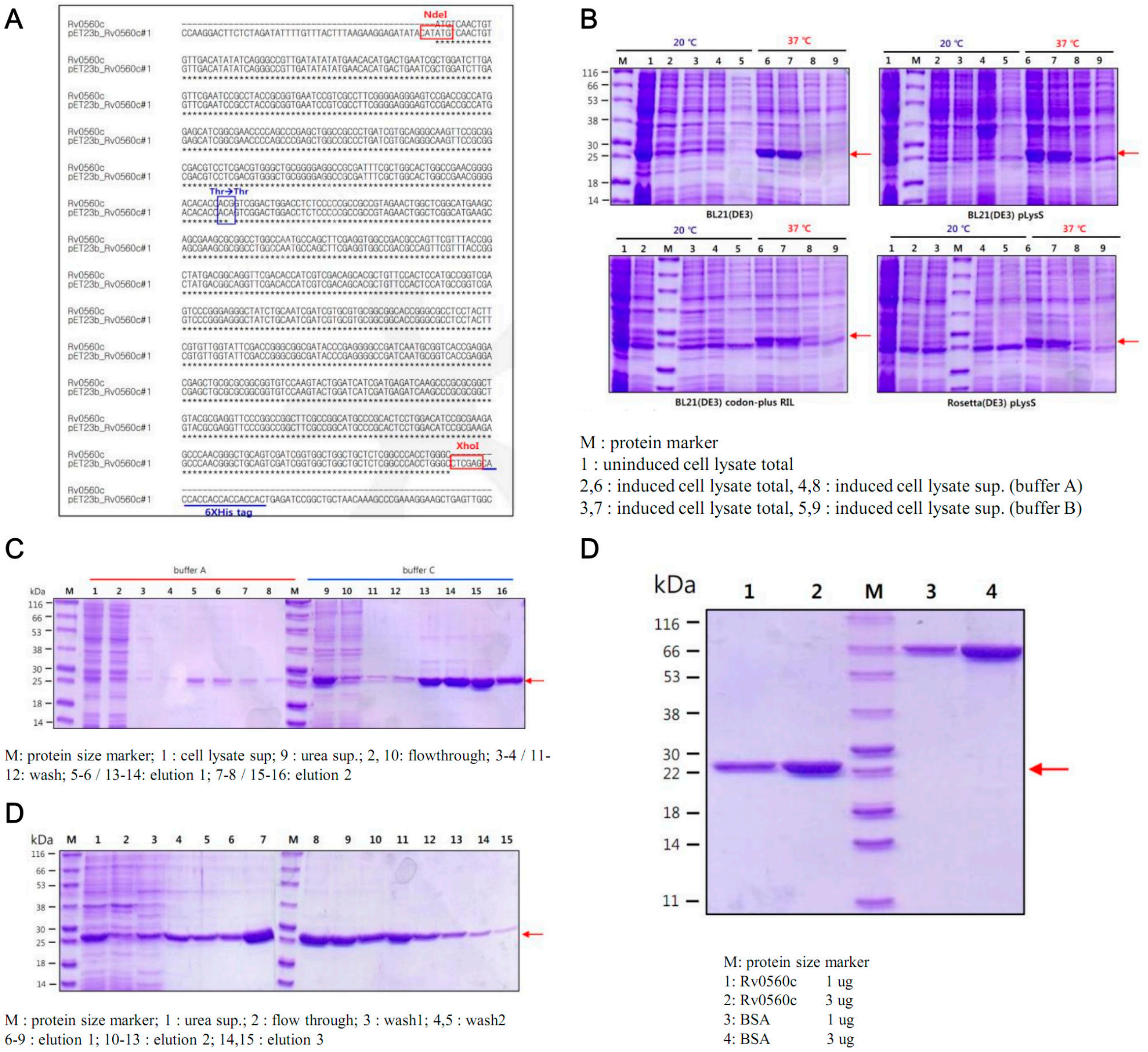

Figure S7. Synthesis of Rv0560c protein. (A) A DNA fragment corresponding to the *Rv0560c* gene amplified using H37Rv gDNA as a template was inserted into the pET23b vector using NdeI/XhoI. (B) Based on the expression test results, a natural binding test was performed using a BL21 (DE3) strain cultured at 37°C. (C) Ni-NTA affinity binding test was performed under native or denaturing conditions. As a result of this experiment, the concentration of the target protein was superior when it was purified under the primary denaturing conditions. (D) Ni-NTA affinity purification was performed under denaturing conditions. (E) Harvested cells were purified and concentrated, and SDS-PAGE confirmed the finally synthesized protein.
